# Knowledge, Practice of Personal Hygiene, School Sanitation, and Risk Factors of Contracting Diarrhea among Rural Students from Five Western Provinces in China

**DOI:** 10.3390/ijerph18189505

**Published:** 2021-09-09

**Authors:** Yu-E Cha, Yuan-Zheng Fu, Wei Yao

**Affiliations:** 1 National Center for Rural Water Supply Technical Guidance, Chinese Center for Disease Control and Prevention, Beijing 102200, China; chayue@nieh.chinacdc.cn; 2 China CDC Key Laboratory of Environment and Population Health, National Institute of Environmental Health, Chinese Center for Disease Control and Prevention, Beijing 100021, China; 18040028047@163.com; 3 Department of Toxicology, School of Public Health, China Medical University, Shenyang 110122, China

**Keywords:** students, sanitation, rural students, knowledge of personal hygiene, practices of personal hygiene, diarrhea

## Abstract

Background: Diarrhea is a global public health issue and a leading cause of childhood malnutrition, growth disturbances, and mortality. The spread of diarrhea is closely linked to the knowledge and maintenance of personal hygiene and quality of drinking water and sanitation facilities. However, there are few such investigations and analysis in rural areas of China. This study aims to determine the association between the risk of contracting diarrhea and knowledge and practices of personal hygiene and school sanitation among rural students as well as provide a scientific basis for preventing the spread of diarrhea and other infectious diseases. A stratified cluster sampling method was used to randomly select 12 rural primary schools in each of 5 counties where the Water, Sanitation and Hygiene (WASH) Plus Program has been implemented. The counties are located in the Guangxi Zhuang autonomous region, Chongqing municipality, Guizhou province, Yunnan province, and Xinjiang Uygur autonomous region. A single fourth-grade class was randomly chosen from each of the 60 schools for observation and a questionnaire survey. The study involved a total of 2330 students. The logistic regression method was adopted to determine the factors contributing to diarrhea in rural students. The results show that male students accounted for 49.40% (n = 1151) of the 2330 research subjects; the average age of the students was 9.9 ± 0.3 years. Approximately 33.09% of the students suffered from diarrhea in the three months leading up to the survey. The odds ratios (ORs) of students who did not know that “diarrhea can be prevented by washing fruits before eating them raw and not drinking untreated water” (OR: 1.303, 95% confidence interval [CI]: 1.063, 1.597) and that “the disease can be prevented by washing hands before meals and after going to the toilet” (OR: 1.522, 95% CI: 1.207, 1.920) were higher than those who knew the above stated facts. Students who “have drunk untreated water at school” (OR: 1.584, 95% CI: 1.268, 1.978), “have drunk untreated water at home” (OR: 1.643, 95% CI: 1.319, 2.048), and “did not wash hands before every meal” (OR: 1.490, 95% CI: 1.120, 1.983) were at a higher risk of contracting diarrhea than those who drank treated water at school and at home and washed their hands before every meal. Diarrhea was more likely to affect students who attended schools with unclean and poorly maintained toilets (OR: 1.586, 95% CI: 1.261, 1.995) or toilets with flies (OR: 1.383, 95% CI: 1.114, 1.717) and without adequate drinking water facilities (OR: 1.407, 95% CI: 1.009, 1.962). The knowledge of methods to maintain personal hygiene, general hygiene practices, and school sanitation are the three major risk factors that account for the spread of diarrhea among rural students from five western provinces (municipalities and autonomous regions) of China. Therefore, to prevent such diseases and maintain health, it is important to provide students with health education, help them develop good hygiene habits, ensure the provision of clean water at schools, and improve the overall school environments.

## 1. Introduction

Diarrhea is defined as a condition in which the patient passes loose or watery stools three or more times per day. It is known to occur commonly in children [1]. In developing and developed countries, diarrhea is mainly caused by viruses, bacteria, and parasites that are transmitted via contaminated food and water [2]. Diarrhea is a global public health issue and a leading cause of childhood malnutrition, growth disturbances, and mortality [3]. Diarrhea has been reported to be the eighth leading cause of mortality in the world, accounting for more than 1.66 million deaths in 2016. Most of the children who succumb to this disease are under the age of 5 years; diarrhea has led to the deaths of 446,000 children in this age group [4]. The incidence and mortality rates of diarrhea remain high among school-aged children, particularly those from low-income countries and regions. Every year, more than 2.8 billion school-aged children suffer from the disease [5]. In China, approximately 836 million people contract infectious diarrhea annually, of whom 678 million are children between 2 and 14 years of age [6]. Diarrhea is among the top five diseases in China with respect to either the mortality rate or disease burden measured in terms of disability-adjusted life years [7]. Schools are densely populated places and provide high-risk environments that facilitate the spread and outbreak of infectious diseases. Research shows that >70% of China’s public health emergencies occur in schools and >80% of school emergencies are triggered by infectious diseases [8]. Zhang et al. [9,10] found a high relative risk of diarrheal disease in western China.

The spread of diarrhea is closely linked to the knowledge and maintenance of personal hygiene and quality of drinking water and sanitation facilities [4,11,12]. For example, washing hands with soap can reduce the risk of the disease by 42–47% and prevent close to 1 million diarrhea-induced deaths [13]. The provision of safe drinking water can further reduce the risk of developing diarrhea by 61%; the provision of adequate sanitation facilities can reduce this risk by an additional 25% [14]. Wang et al. [6] reported a large gap between low-income countries and high-income countries in terms of the burden of diarrhea, which is mainly calculated in low-income countries based on the number of premature deaths and loss of the ability to work. Globalization has drastically increased the frequency of movement of people across the world. Therefore, a global effort is needed to curb the increasing burden of diarrhea by determining its risk factors and controlling its spread, along with carrying out the method of health literacy co-design in rural areas to improve health and equity of rural students [15]. Between 2016 and 2020, the Ministry of Education of the People’s Republic of China and the United Nations International Children’s Emergency Fund (UNICEF) implemented the WASH Plus Program (hereafter referred to as “the Program”) at 300 rural primary schools in five provinces (municipalities or autonomous regions) in underdeveloped regions of western China. The Program aimed to provide safe drinking water and improve sanitation facilities (toilets and hand washing facilities) for children at the selected schools, thus helping them develop good hygiene practices [16]. Based on cross-sectional survey data from the Program obtained in 2018, we analyzed the associations between the contraction of diarrhea and students’ knowledge and practice of personal hygiene and school sanitation conditions.

## 2. Methods

### 2.1. Respondents

**Figure 1:**
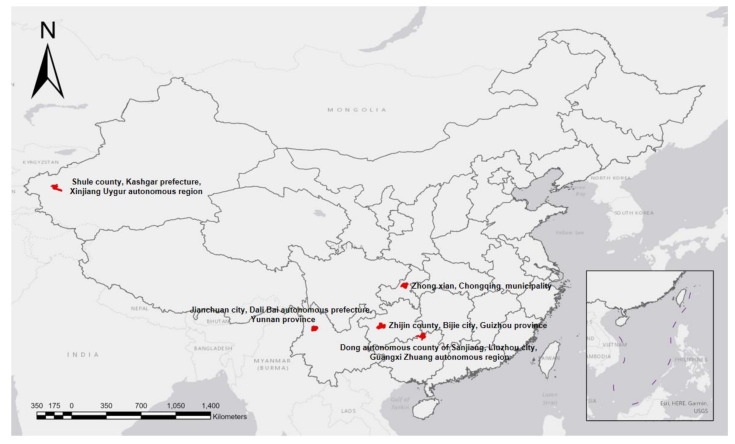
Locations of provinces where the study was conducted.

A stratified cluster sampling method was used to randomly select five counties (one county in each of the five provinces (municipalities or autonomous regions), i.e., Guangxi Zhuang autonomous region, Chongqing municipality, Guizhou province, Yunnan province, and Xinjiang Uygur autonomous region) where the Program was implemented. These regions are highlighted in Figure 1.

The researchers randomly selected a single fourth-grade class from each of the 60 primary schools in the five counties (12 schools in each county). If a chosen class had <30 students, fifth-grade students were randomly selected to meet the quota requirement. A total of 2330 students participated in the survey. We use this formula t$\;n=\frac{\mu_{\alpha }^{2}\times \;\pi \;\times \left(1-\pi \right)}{\delta^{2}}\;$o to calculate the sample size (n): *α* = 0.05, *μ_α_* = 1.96, π is incidence rate of diarrhea is 10%, and the error rate is 3%. *δ*^2^ = 3% × 3% = 0.0009), the lost follow-up rate was 5%, and the sample size was about 405. Each province surveyed at least 405 students.

### 2.2. Survey Method

From September to December 2018, the researchers visited the 60 selected schools and asked students to fill out an anonymous questionnaire by themselves. Researchers explained the survey questions to the students before conducting the survey, checked their answers for completeness, and examined their hand hygiene and fingernail length when collecting the questionnaires. The questionnaire included questions about the students’ demographics, hygiene knowledge, hygiene practices, school sanitation conditions, and whether they had had diarrhea in the 3 months prior to the survey period. All of the 2330 questionnaires distributed were returned and deemed valid. The teachers and students fully understood the survey content and agreed to participate in the survey, which was agreed on by the parents of the students before the survey. During the survey, the teachers were present in the whole process, and obtained the relevant health knowledge, attitude and behavior information through the students’ self-administered questionnaire. The whole process did not cause any harm to the students, and they exchanged ideas. There is no risk to the target. Before the investigation, we strictly implemented the requirements of informed consent in the process of investigation. Through the review of information and on-site investigation according to the ethical review provisions, the project met the ethical requirements, and it has been proved by National Center for Rural Water Supply Technical Guidance, Chinese Center for Disease Control and Prevention.

### 2.3. Quality Control

The research subjects were randomly selected in strict accordance with related technical procedures. The questionnaire was subjected to expert consultation and pre-testing. The research team, which consisted of professional technicians, answered any questions raised by the students on-site and examined the completeness of their responses. Double data entry and real-time validation were used to ensure the accuracy of the data.

### 2.4. Statistical Analysis

The researchers used Microsoft Excel 2016 for data entry and sorting and the Statistical Package for Social Sciences (SPSS) v19.0 for statistical analysis. Quantitative variables are represented as means ± standard deviations (SD). Grouped data are expressed as percentages. Univariate analysis was performed with Chi-square test for diarrhea, and multiple logistic regression analysis was conducted with statistically significant factors and some potentially significant factors in univariate analysis as independent variables. Adjusted odds ratios (ORs) and their 95% confidence intervals (CIs) were computed. Two-tailed tests were carried out at an alpha level of 0.05.

## 3. Results

### 3.1. Basic Information

The 2330 respondents (aged 9.9 ± 0.3) years consisted of 461 students from Guangxi Zhuang autonomous region, 538 from Chongqing municipality, 465 from Guizhou province, 410 from Yunnan province, and 456 from Xinjiang Uygur autonomous region. Of these, 1151 students (49.40%) were male, 1489 (63.91%) were of a minority ethnicity, and 433 (18.58%) were boarding students. Approximately 771 students (33.09%) suffered from diarrhea in the 3 months leading up to the survey; 32.67% of students in this subset were male (n = 376) and 33.50% were female (n = 395). Among the diarrhea-afflicted students, 175 were from Guangxi Zhuang autonomous region, 88 from Chongqing municipality, 156 from Guizhou province, 175 from Yunnan province, and 177 from Xinjiang Uygur autonomous region, accounting for 37.96%, 16.36%, 33.55%, 42.68%, and 38.82% of the total respondents from these regions, respectively. Approximately 24.02% (n = 202) and 38.21% (n = 569) of students of Han and minority ethnicity, respectively, contracted diarrhea, and 35.57% (n = 154) and 32.53% (n = 617) of boarding and non-boarding students, respectively, were found to have had the disease. The demographic information is outlined in Table 1.

### 3.2. Univariate Logistic Regression Analysis

A chi-square test was carried out to analyze factors that may contribute to the spread of diarrhea in students, such as their basic information, hygiene knowledge, hygiene practices, and school sanitation conditions. Of the four basic information factors, “ethnic group” and “region” were found to be statistically significant (*p* < 0.05). Approximately 38.21% of students of minority ethnicity had suffered from the disease, compared to 24.02% of students of Han ethnicity. The survey showed that 42.68% of students from Yunnan Province had suffered from diarrhea, and that this region had the highest diarrhea rate among all five provinces.

Of the nine factors related to hygiene knowledge, only the factor of “whether students know that diarrhea can be transmitted through contaminated water” was statistically insignificant (*p* > 0.05). Students who did not possess adequate hygiene knowledge were more vulnerable to diarrhea than those who did possess such knowledge. Close to 50.38% of students who did not know the “the right way to wash their hands,” 47.19% of students who did not know that “open defecation may pollute water sources, damage environmental sanitation, and breed mosquitoes and flies,” 46.54% of students who did not know that “hand washing can prevent diseases like diarrhea, dysentery, hepatitis A, and roundworm disease,” 45.81% of those who were unaware of that “diarrhea can be prevented by washing hands before meals and after going to the toilet,” and 45.78% of students who did not know that “dirty hands can transmit diseases” had all suffered from diarrhea in the 3 months leading up to the survey.

All of the 11 hygiene practice factors were found to be statistically significant (*p* < 0.05). Students who did not practice hygiene were more susceptible to the disease than those who did. The diarrhea rates were high among students who “did not wash vegetables and fruits every time before eating them raw” (52.16%), those who “did not like health classes” (51.28%), those who “did not wash hands before every meal” (50.28%), those who “did not wash hands after every visit to the toilet at home” (49.60%), those who “have drunk untreated water at school” (48.27%), those who “have drunk untreated water at home” (47.53%), and those who “did not wash hands after every visit to the toilet at school” (47.53%).

With respect to the seven school sanitation factors, only the factor of “whether students have to queue for toilets” was statistically insignificant (*p* > 0.05). Students were more likely to contract diarrhea if they had “no hand washing or drinking water facilities at school,” “had to queue for toilets at school,” or reported that “the toilets at school were unsafe with flies” and “the toilets smelled bad.” Approximately 52.33% of students in “schools with unsafe toilets,” 49.72% of those in “schools without hand washing facilities,” and 48.22% of those in “schools without drinking water facilities” had suffered from diarrhea in the 3 months leading up to the survey.

All of the above results are outlined in Table 2.

### 3.3. Multiple Logistic Regression Analysis

A history of diarrhea in the 3 months leading up to the survey was designated as the dependent variable (Y: 0 = No diarrhea, 1 = Diarrhea), and the aforementioned 27 statistically significant factors were included in the unconditional logistic regression model for analysis. We found that students from Yunnan province were at greater risk of contracting diarrhea than those from Chongqing municipality (OR: 2.05, 95% CI: 1.34, 3.14). Students who did not know that “diarrhea can be prevented by not drinking untreated water and by washing fruits before eating them raw” (OR: 1.303, 95% CI: 1.063, 1.597) and did not know that “the disease can be prevented by washing hands before meals and after going to the toilet” (OR: 1.522, 95% CI: 1.207, 1.920) were more susceptible to diarrhea than those who knew the above-stated facts. Students who “have drunk untreated water at school” (OR: 1.584, 95% CI: 1.268, 1.978), “have drunk untreated water at home” (OR: 1.643, 95% CI: 1.319, 2.048), and “did not wash hands before every meal” (OR: 1.490, 95% CI: 1.120, 1.983) were at higher risk than those who followed the above-described hygiene practices. Diarrhea was more likely to affect students who attended schools with poorly maintained and dirty toilets (OR: 1.586, 95% CI: 1.261, 1.995), toilets that had flies (OR: 1.383, 95% CI: 1.114, 1.717), and inadequate drinking water facilities (OR: 1.407, 95% CI: 1.009, 1.962). The results of this analysis are outlined in Table 1.

## 4. Discussion

The results of our study show that 33.09% of the research subjects had suffered from diarrhea in the 3 months leading up to the survey. Victoria et al. conducted a survey in Mali on 4907 fourth-grade students [17] with an average age of 11 years who were subjected to WASH interventions and 4823 similarly aged students who were not subjected to such interventions; they found that 10.0% and 13.0% of the students, respectively, had contracted diarrhea in the week preceding the investigation. A study conducted by Emma et al. showed that 11% of 2082 students (fourth- and fifth-graders, aged 10 years on average) surveyed from 116 schools across eight states and regions in Myanmar had contracted diarrhea in the week leading up to the survey [18]. Mansour et al. discovered that 14.9% of 1064 children surveyed in Saudi Arabia had contracted the disease in the month preceding the survey [19]. Other studies have shown that 16.4% [20], 14.42% [21,22], and 12.1% of children under the age of 5 years in Ethiopia, Indonesia, and Tanzania [23], respectively, had contracted diarrhea in the 2 weeks preceding the study. All of the above-described studies focused on diarrhea in children under the age of 5 years and on a time period of 1 month before the survey. Mahmud et al. found that the intestinal parasite reinfection rates were 29%, 26%, and 38% in children who had not been subjected to the hand washing intervention, children whose fingernails were not cut, and children who had been subjected to neither of the interventions, respectively [22]. These results are consistent with those of our study.

We found that the number of students suffering from diarrhea in Yunnan province was higher than that in the other four regions (1.6 times that in Chongqing municipality). Liu M et al. showed that residents in Yunnan province did not perform well when tested for basic knowledge about health, good health practices, and skills for maintaining health [24]. They also argued that ethnic minorities were in greater need of improvements in health literacy. A study conducted by Zhou Y et al. showed that between 2011 and 2013, there were 39,347 reports of diarrhea from Yunnan province, amounting to an average incidence rate of 28.34/100,000 [25]. Furthermore, the province saw an upward trend in reported cases during this 3-year period. These data imply that to prevent the spread of diarrhea and other contagious diseases among rural students, more targeted health education and effective WASH interventions should be implemented.

The univariate logistic regression analysis showed that eight of nine factors related to hygiene knowledge were statistically significant (*p* < 0.05), suggesting that health education plays a key role in preventing the spread of infectious diseases such as diarrhea. The multiple logistic regression analysis indicated that a lack of the knowledge that “diarrhea can be prevented by washing fruits before eating them raw and not drinking untreated water” and “diarrhea can be prevented by washing hands before meals and after going to the toilet” were the two major risk factors associated with the disease. Students who were unaware of these two health factors had 1.32 and 1.54 times the risk for diarrhea, respectively, when compared with those who knew about and practiced them. Intestinal pathogens responsible for diarrhea are mainly transmitted through contaminated food and water by the fecal–oral route. Therefore, simple interventions, such as drinking boiled water, are very effective against the spread of diarrhea [14,26]. The outbreak of COVID-19 has attracted increased attention to the fecal–oral transmission of diseases. Viruses present in patient feces can be transmitted to others through food from fields contaminated by feces, food exposed to contaminated water, or objects, hands, and flies contaminated by virus-laden feces [27]. Unwashed hands are a main entry point for both viruses and bacteria. The proper hand washing guidelines laid out by the World Health Organization involve the use of soap and water, and include six steps to ensure that hands are thoroughly washed [28]. Hand washing helps to reduce the overall biomass of microbes, thus preventing diarrhea and respiratory infections as well as reducing the odds of resultant absences from school [29].

This study found 11 factors related to hygiene practices that were all statistically significant, implying that good hygiene practices are key to the prevention of infectious diseases such as diarrhea. The multiple logistic regression analysis indicated that “have drunk untreated water at school”, “have drunk untreated water at home”, and having not “washed hands before every meal” are the three major risk factors associated with diarrhea. Students who exhibited the above three behaviors faced 1.57, 1.54, and 1.43 times the risk, respectively, of contracting the disease than those who followed good hygiene practices. An epidemiological study on an infectious diarrhea outbreak in a school in Zhenba county in central Shaanxi province, China, reported that the outbreak was mainly triggered by students drinking dirty water [30]. Xiao et al. reported that “never washing vegetables and fruits/washing them occasionally before eating them raw” and “never washing hands/washing hands occasionally before meals and after going to the toilet” contribute to significant risks of intestinal parasite infections [31].

In addition to the above, the availability of hand washing and drinking water facilities and the conditions of toilets at schools are important factors that determine students’ risk of contracting diarrhea. The multiple logistic regression analysis showed that schools without adequate drinking water facilities, with ill-maintained and unclean toilets, or with fly-ridden toilets posed significant hidden dangers for students. Students from schools that met these conditions faced 1.42, 1.5, and 1.34 times the risk, respectively, of contracting diarrhea than those who studied at schools with better sanitation conditions. The sanitation conditions of schools play a significant role in the development of good hygiene practices, as most children learn about such practices during their formal education [32]. If hand washing and adequate drinking water facilities are unavailable at schools, students cannot wash their hands properly and are more likely to drink untreated water [33]. A study conducted by Hughes et al. with Pacific Island communities found that students attending schools without adequate drinking water supplies faced a risk of contracting helminthiasis that was four times as high as that of students in schools with good water supply systems [34]. Students lacking access to safe water and sanitation are highly susceptible to infectious diseases, gastrointestinal problems, neurocognitive disorders, and mental health problems [35]. Recent research shows that 51% of students avoid using school toilets [36] because the toilets are either dirty or unsafe [37]. Holding in urine/stool for long periods of time increases the risk of incontinence, constipation, and urinary tract infections [18]. Students with access to clean toilets at schools are also 12.17% less likely to suffer from diarrhea than those who lack access to such facilities [18].

Through the analysis of this study, it is concluded that the knowledge of methods to maintain personal hygiene, general hygiene practices, and school sanitation are the three major risk factors that account for the spread of diarrhea among rural students from five western provinces of China. Moreover, the number of students suffering from diarrhea in Yunnan province was higher than that in the other four regions.

Based on the risk factors associated with contracting diarrhea in students, we propose the following four suggestions to limit spread of the disease. First, health education must be prioritized and WASH interventions must be applied to vulnerable groups and poverty-stricken areas, especially Yunnan. Second, health education programs should be implemented in schools to raise awareness about sanitation and disease prevention; the programs should address topics such as the routes of disease transmission and help students develop good hygiene practices to protect themselves. Third, elements of the WASH intervention, such as conducting sanitation-themed lectures and health classes, distributing promotional materials advocating good health practices, and helping students stop bad hygiene practices and adopt good ones, such as not drinking untreated water, washing hands before meals and after using the toilet, washing raw fruits thoroughly before eating them, trimming fingernails often, and washing hands with running water and soap/hand sanitizer should be systematically implemented. Fourth, the water supply and sanitation conditions in schools should be improved; this is key to preventing diseases and keeping students healthy. Schools should either provide safe and clean drinking water facilities for students or mandate that students bring safe drinking water from home to prevent them from drinking untreated water. Hand washing facilities must be available to allow students to wash their hands before meals and after using the toilets at school. Schools should renovate unsafe and unhygienic toilets and teach students how to use a toilet properly; at the very least, they should ensure that the toilets are cleaned regularly and do not have flies. However, it is the implementation of these recommendations that may present challenges. Health literacy co-design can be carried out in rural areas to use local wisdom to inform interventions for rural students to improve health and equity [15].

The limitations of this study are as follows: 1. The investigation of diarrhea is not based on medical records, but based on the questionnaire, and the results may have memory bias. 2. This study did not include all age groups of primary school students, only including the fourth grade students. The extrapolation of the relationship between diarrhea rate and environmental health knowledge and behavior of primary school students in different age groups was limited. 3. Due to the special research group and limited by the privacy of the questionnaire, this study did not consider other potential confounding factors such as socioeconomics, demographics, and other environments.

## 5. Conclusions

The knowledge of methods to maintain personal hygiene, general hygiene practices, and school sanitation are the three major risk factors that account for the spread of diarrhea among rural students from five western provinces (municipalities and autonomous regions) of China. Therefore, to prevent such diseases and maintain health, it is important to integrate the multi sectoral resources by government departments, such as education, health, Poverty Alleviation Office, agriculture and other departments, to further guide the work related to school water and environmental sanitation, make full use of domestic project funds and UNICEF project funds to bundle implementation, complement each other and develop together in order to provide students with health education, help them develop good hygiene habits, ensure the provision of clean water at schools, and improve the overall school environments.

## Figures and Tables

**Table 1:** Demographics of study participants (N = 2330).

Demographics	Number of Subjects (% Subject Population) or Mean ± SD
Age (years)	9.9 ± 0.3
Sex	
Male	1151 (49.40)
Female	1179 (50.60)
Ethnic group	
Han people	841 (36.09
Ethnic minority	1489 (63.91)
Boarding	
Yes	433 (18.58)
No	1897 (81.42)
Region	
Chongqing municipality	538 (23.09)
Guizhou province	465 (19.96)
Guangxi Zhuang autonomous region	461 (19.79)
Xinjiang Uygur autonomous region	456 (19.57)
Yunnan province	410 (17.60)

**Table 2:** Univariate and multiple logistic regression analysis of factors associated with the risk of contracting diarrhea.

Item	Category	Univariate Logistic Regression Analysis					Multiple Logistic Regression Analysis				
		Number of Respondents	Number of Students Who Contracted Diarrhea	Diarrhea Rate (%)	χ^2^ Value	*p* Value	β Value	*SE*	Wald χ^2^ Value	OR (95% CI)	*p* Value
**Basic Inform-ation**	Sex				0.184	0.668	–	–	–	–	–
	Male	1151	376	32.67			–	–	–	–	–
	Female	1179	395	33.50			–	–	–	–	–
	Ethnic group				48.909	0					
	Han people	841	202	24.02						1.00	
	Ethnic minority	1489	569	38.21			0.040	0.150	0.071	1.04 (0.78, 1.40)	0.790
	Boarding				1.472	0.225	–	–	–	–	–
	Yes	433	154	35.57			–	–	–	–	–
	No	1897	617	32.53			–	–	–	–	–
	Region				96.815	0.000					
	Chongqing municipality	538	88	16.36						1.00	
	Guizhou province	465	156	33.55			0.357	0.187	3.627	1.43 (0.99, 2.06)	0.057
	Guangxi Zhuang autonomous region	461	175	37.96			0.249	0.222	1.262	1.28 (0.83, 1.98)	0.261
	Xinjiang Uygur autonomous region	456	177	38.82			0.153	0.232	0.432	1.17 (0.74, 1.84)	0.511
	Yunnan province	410	175	42.68			0.717	0.217	10.887	2.05 (1.34, 3.14)	0.001 *
**Hygiene know-ledge**	Drinking untreated water is harmful to health.				29.504	0.000					
	Aware	1887	576	30.52						1.00	
	Unaware	443	195	44.02			0.032	0.127	0.064	1.03 (0.81, 1.33)	0.801
	Diarrhea can be transmitted through contaminated water.				0.959	0.328	-	-	-	-	-
	Aware	973	311	31.96			-	-	-	-	-
	Unaware	1357	460	33.90			-	-	-	-	-
	Dirty hands can transmit diseases.				20.287	0.000					
	Aware	2081	657	31.57						1.00	
	Unaware	249	114	45.78			0.113	0.155	0.539	1.12 (0.83, 1.52)	0.463
	The correct way to wash hands.				19.036	0.000					
	Aware	2197	704	32.04						1.00	
	Unaware	133	67	50.38			0.148	0.208	0.507	1.16 (0.77, 1.74)	0.476
	Hand washing can prevent diseases like diarrhea, dysentery, hepatitis A, and roundworm disease.				13.944	0.000					
	Aware	2171	697	32.11						1.00	
	Unaware	159	74	46.54			0.097	0.199	0.238	1.1 (0.75, 1.63)	0.626
	Feces can transmit diseases like roundworm disease, hookworm disease, diarrhea, and dysentery.				8.060	0.005					
	Aware	1974	630	31.91						1.00	
	Unaware	356	141	39.61			−0.093	0.141	0.434	0.91 (0.69, 1.20)	0.510
	Diarrhea can be prevented by washing fruits before eating them raw and not drinking untreated water.				26.466	0.000					
	Aware	1605	477	29.72						1.00	
	Unaware	725	294	40.55			0.276	0.105	6.939	1.32 (1.07, 1.62)	0.008 *
	Diarrhea can be prevented by washing hands before meals and after going to the toilet.				41.238	0.000					
	Aware	1876	563	30.01						1.00	
	Unaware	454	208	45.81			0.432	0.119	13.096	1.54 (1.22, 1.95)	0 *
	Open defecation may pollute water sources, damage environmental sanitation, and breed mosquitoes and flies.				8.310	0.004					
	Aware	2241	729	32.53						1.00	
	Unaware	89	42	47.19			−0.017	0.254	0.005	0.98 (0.60, 1.62)	0.946
**Hygiene practices**	I never drink untreated water at school.				143.404	0.000					
	Yes	1464	353	24.11						1.00	
	No	866	418	48.27			0.451	0.115	15.344	1.57 (1.25, 1.97)	0 *
	I never drink untreated water at home.				131.024	0.000					
	Yes	1459	357	24.47						1.00	
	No	871	414	47.53			0.432	0.115	14.111	1.54 (1.23, 1.93)	0 *
	I wash vegetables and fruits every time before eating them raw.				47.016	0.000					
	Yes	2075	638	30.75						1.00	
	No	255	133	52.16			0.293	0.158	3.419	1.34 (0.98, 1.83)	0.064
	I wash hands before every meal.				56.449	0.000					
	Yes	1972	591	29.97						1.00	
	No	358	180	50.28			0.358	0.147	5.962	1.43 (1.07, 1.91)	0.015 *
	I wash hands after every visit to the toilet at school.				27.914	0.000					
	Yes	2067	646	31.25						1.00	
	No	263	125	47.53			0.061	0.167	0.135	1.06 (0.77, 1.47)	0.713
	I wash hands after every visit to the toilet at home.				34.800	0.000					
	Yes	2078	646	31.09						1.00	
	No	252	125	49.60			−0.068	0.174	0.153	0.93 (0.66, 1.31)	0.696
	I wash hands with running water and soap/hand sanitizer at school.										
	Yes	1583	471	29.75	24.826	0.000				1.00	
	No	747	300	40.16			0.014	0.117	0.015	1.01 (0.81, 1.28)	0.903
	I wash hands with running water and soap/hand sanitizer at home.				28.771	0.000					
	Yes	1793	542	30.23						1.00	
	No	537	229	42.64			0.040	0.121	0.111	1.04 (0.82, 1.32)	0.739
	I like health classes.				24.991	0.000					
	Yes	2174	691	31.78						1.00	
	No	156	80	51.28			0.128	0.195	0.433	1.14 (0.78, 1.67)	0.511
	My hands are clean.				24.465	0.000					
	Yes	1848	566	30.63						1.00	
	No	482	205	42.53			0.042	0.122	0.121	1.04 (0.82, 1.33)	0.728
	I have long fingernails.				6.786	0.009					
	No	1789	567	31.69						1.00	
	Yes	541	204	37.71			0.117	0.118	0.985	1.12 (0.89, 1.42)	0.321
**School sanitation**	School has hand washing facilities.				23.919	0.000					
	Yes	2153	683	31.72						1.00	
	No	177	88	49.72			0.150	0.187	0.648	1.16 (0.81, 1.68)	0.421
	School has drinking water facilities.				22.259	0.000					
	Yes	2133	676	31.69						1.00	
	No	197	95	48.22			0.351	0.171	4.239	1.42 (1.02, 1.99)	0.040 *
	School toilets are clean.				34.117	0.000					
	Yes	1726	513	29.72						1.00	
	No	604	258	42.72			0.110	0.129	0.727	1.12 (0.87, 1.44)	0.394
	I have to queue for toilets.				1.839	0.175					
	No	1537	494	32.14							
	Yes	793	277	34.93							
	Toilets smell bad.				94.187	0.000					
	No	969	212	21.88						1.00	
	Yes	1361	559	41.07			0.404	0.120	11.341	1.5 (1.18, 1.89)	0.001 *
	There are flies in the toilets.				96.381	0.000					
	No	1101	253	22.98						1.00	
	Yes	1229	518	42.15			0.290	0.113	6.550	1.34 (1.07, 1.67)	0.010 *
	The toilets are safe.				14.923	0.000					
	Yes	2244	726	32.35						1.00	
	No	86	45	52.33			0.260	0.245	1.131	1.30 (0.8, 2.10)	0.288

* Indicates that the significance level in the multiple logistic regression analysis is *p* < 0.05. OR: odds ratio; CI: confidence interval.
